# State of emergency medicine in Colombia

**DOI:** 10.1186/s12245-015-0057-4

**Published:** 2015-04-10

**Authors:** Christian Arbelaez, Andrés Patiño

**Affiliations:** Harvard Affiliated Emergency Medicine Residency Program, Brigham and Women’s Hospital, 75 Francis St., Boston, MA 02115 USA; Harvard Affiliated Emergency Medicine Residency Program, Brigham and Women’s Hospital/Massachusetts General Hospital, 75 Francis St., Boston, MA 02115 USA

**Keywords:** Colombia, EM, International EM development, Residency education

## Abstract

Colombia is an upper-middle-income country with a population of 45 million people and one of the best national healthcare and medical education systems in South America. However, its widely diverse and difficult terrains hinder healthcare delivery to rural areas, creating disparities in healthcare access and outcomes between the urban and rural settings. Currently, emergency medical care is overwhelmingly provided by general practitioners without residency training, who obtain specialty consultations based on the medical/surgical condition identified. A few emergency medicine (EM) residency programs have sprouted over the last two decades in renowned academic institutions in the largest cities, producing high-quality EM specialists. With the establishment of EM as a specialty in 2005 and increasing recognition of the specialty, there has been an increasing demand for EM specialists in cities, which is only slowly being met by the current residencies. The critical challenges for EM in Colombia are both, establishing itself as a well-recognized specialty - by increasing academic production and reaching a critical mass of and unity among EM specialists - and providing the highest quality and safest emergency care to the people of Colombia - by improving capacity both in emergency departments and in the regional and national emergency response systems. Historically, the establishment of EM as a strongly organized specialty in other countries has spanned decades (e.g., the United States), and Colombia has been making significant progress in a similar trajectory.

## Background

### Introduction

Colombia covers an area of 1.1 million square miles in northern South America with a population of 45 million in 2010 [1]. Seventy-five percent of the population lives in cities, and the rest live in a variety of terrains [2]. Bogota is the capital and largest city, followed by Medellin and Cali. Colombia’s government is a presidential republic with state administration decentralized among 32 departments [1]. Colombia is an upper-middle-income country [3], and its economy saw sustained growth in the last decade with an average annual growth in GDP of 3.3% from 2006 to 2010 [4] and a GDP per capita in 2009 of US$ 4,990 [1]. Despite this growth, corruption, unemployment, underemployment, job insecurity, and inequality persist. Unemployment between 2006 and 2010 remained at 11% [1]. Forty-six percent of the population lived in poverty and 16.4% in extreme poverty, and the top 20% of the population held 25 times the wealth of the poorest 20% in 2009 [1]. Social inequalities, isolation of rural communities due to difficult terrains, and the development of the drug trade have served as fuel for one of the world’s oldest armed conflicts. For over 50 years, the Colombian government has been fighting guerrilla groups, namely, the Revolutionary Armed Forces of Colombia (FARC), the National Liberation Army (ELN), and United Self Defense Forces of Colombia (AUC) (now demobilized), as well as drug cartels and smaller drug-trafficking groups. The conflict has disproportionally affected agricultural communities where the presence of the state is weakest or nonexistent, resulting in an estimated 5.7 million internally displaced people [5]. Though insurgent groups lack the military or popular support necessary to overthrow the government, they are heavily funded by the drug trade and continue to attack civilians and retain influence over large areas of the Colombian countryside [2]. Despite this internal conflict, Colombia has managed to maintain relatively strong democratic institutions with peaceful elections and protection of civil liberties [2]. Over the last 10 years, improvements in security and strong economic growth have positioned Colombia as an emerging market and an increasingly popular travel destination.

### National healthcare system

As an upper-middle-income country, Colombia has a unique dichotomy of healthcare. In urban settings, the healthcare system can be one of the best in the continent with preeminent hospitals and training programs comparable to those in the US. In stark contrast, in most rural areas, including some of the most remote locations in the world like the Amazon forest, a frail and fragmented healthcare system prevails, similar to those in third world countries, with limited resources and lack of healthcare providers. Infrastructure problems are compounded by factors such as socioeconomic determinants of health, barriers to care, displaced and vulnerable refugee populations impacted by violence, and a diverse terrain. Furthermore, Colombia is among the countries most susceptible to natural disasters, including floods, earthquakes, and volcanic eruptions, which makes rural populations even more at risk of disease. This dichotomy in healthcare settings causes a wide gap in the life expectancy, leading causes of death, and overall healthcare outcomes in the Colombian population, gaps which are often seen in the emergency department and can be exponentially impacted, positively or negatively, by the quality of emergency care delivered. In Colombia, life expectancy for men is 74 years and for women is 81 years [3]. After adjusting for age, homicides are the leading cause of death accounting for 22%, followed by neoplasms, ischemic heart disease, acute respiratory infections, and cerebrovascular diseases [6].

In Colombia, as is the case in most countries, the public and private sectors finance healthcare creating a two-tiered healthcare delivery system whereby those who can afford care and those who cannot receive it in very different levels. All employees in Colombia are mandated to purchase private health insurance from companies called *Empresas Promotoras de Salud* (EPS). Employers are mandated to match their employees’ payments to their EPS. Monthly EPS premiums are dictated by a universal sliding scale, based on income, as mandated by the government, and a given fee provides coverage for the employee and his/her dependents (spouse and children), regardless of the number of dependents. The poorest segment of the population qualifies for similar private insurance paid by the government. Qualification for subsidized insurance is determined by an application called SISBEN. A large number of private hospitals, known as *clínicas*, provide care for most insured patients, are usually well-resourced, and include world-class tertiary/quaternary centers. Yet, there is a large segment of the population that remains uninsured in a gap between the two forms of coverage. The population in this gap tends to be poor but not poor enough to qualify for subsidized insurance through SISBEN, works informal jobs (that do not comply with the law mandating health insurance coverage for employees), and cannot afford or chooses not to pay private insurance. This population ends up paying out-of-pocket for their healthcare or seeking care in public hospitals. Colombia has a network of public hospitals serving both the insured and uninsured, ranging from small clinics and hospitals in rural areas to referral centers in larger cities, most of which tend to have very limited resources as they rely on government funding to provide care for the uninsured.

In remote rural areas of Colombia, there is a significant lack of access to medical care, especially in hard to reach areas affected by natural disasters. Further complicating this is the reality that many of these isolated medical facilities are understaffed, often with only a nurse or nurse’s aide, and are often closed due to these staffing constraints. If a patient with an emergent medical condition requires transfer to a higher level of care, the distance and modes of transportation (e.g., canoe, horseback, or motorcycle) can be challenging and often lead to higher morbidity and mortality. Often, patients from rural areas are taken to the next highest level of care available before reaching a hospital, often a clinic in a small rural town, in order to receive some stabilization by a physician, most often a recent medical graduate. Colombian medical graduates are required to work in public service or in a rural area for 1 year after graduating medical school and constitute a safety net to some of the most vulnerable populations in remote locations.

### Medical education

Students who are interested in Medicine apply to medical school immediately after graduating high school. Admissions are competitive and based on scores in the ICFES, a national, standardized college admission test. Medical school lasts 6 to 7 years and includes internship as its last year (the equivalent of post-graduate year 1 in the US). Medical graduates are then required by the government to complete a public service, or a year in a rural area, working in underserved communities as a general practitioner, as mentioned above. After this year, the majority of medical graduates go on to work as general practitioners in the community or work under the supervision of specialists in tertiary academic centers. Residency spots in all specialties are very competitive. Medical graduates apply to each program individually as there is no centralized application system. Applications usually include written exams, followed by interviews for those with the best scores. The great majority of those selected do not receive compensation during residency and instead have to pay tuition. In Colombia, there are residency programs in all specialties and most subspecialties, including emergency medicine (Table 1). With its specialization over the last decade, the delivery of emergency care within this healthcare system continues to grow, develop, and transform the pre-hospital and hospital components of emergency medicine.

**Table 1:** **Emergency medicine residency programs in Colombia**

**Program**	**CES**	**Rosario**	**Antioquia**	**Javeriana**	**FUCS**	**Fundacion Valle del Lilly and HUV**
City	Medellin	Bogota	Medellin	Bogota	Bogota	Cali
Year started	1996	2001	2004	2008	2008	2014

### Emergency care system

The delivery of emergency care is best evaluated in the two phases of pre-hospital and hospital care. The pre-hospital system is a combination of the Anglo-French and the American emergency medical systems. Depending on the geographical location, the pre-hospital transport modalities range from volunteer community members who drive patients by private car or motorcycle, horseback in remote mountainous locations, or canoe in areas accessible only by water, to critical care transport units, including ambulances and fixed wing planes. Most ambulances are privately owned and are staffed by emergency medical technicians or general practitioners. Advanced air and ground transport services for transfer of critically ill patients to major referral centers are staffed by medical teams with specialty trained physicians, which may include emergency physicians. The pre-hospital system in Colombian cities is usually well developed and provides a key first link for emergency care.

Emergency hospital care is tiered based on available resources and technical capabilities of each facility. At the lower levels, typically remote rural sites, clinics, or small hospitals have limited resources and personnel who stabilize and transfer patients to a higher level of care. A mix of public and privately funded clinics/hospitals with emergency departments constitute the higher level facilities, mostly found in metropolitan areas, where general practitioners see low acuity patients independently and triage high acuity patients to the appropriate specialist. Emergency medicine specialists are still few and tend to work in large emergency departments seeing critically ill patients. As the specialty of emergency medicine continues to grow and meet the current demand for highly trained emergency physicians, the quality and safety of emergency care should continue to improve in Colombia.

### Emergency medicine specialty

Colombia has been at the vanguard of creating and consolidating emergency medicine as a specialty in Latin America. In 1996, CES University created the first emergency medicine residency program in Medellin, a 3-year program that has now graduated the most EM specialists in Colombia. In 2001, University of Rosario in conjunction with one of the largest and most technologically advanced private hospitals, Clínica Santa Fe de Bogotá, founded the second EM residency training program, as the first 4-year program. In 2004, the University of Antioquia in Medellin developed a 3-year program and became the first public university to offer training in the specialty. A major landmark for the specialty occurred in 2005 when the Colombian Ministry of Social Protection (Ministry of Health) recognized emergency medicine as a specialty. In 2008, Javeriana University and FUCS University started EM residency programs in Bogota, 3 and 4 years in length, respectively. Most recently in 2014, in the first public-private partnership, Fundacion Valle del Lili with Hospital Universitario del Valle (HUV) received approval to start a residency program in Cali. Currently, there are other universities requesting to start EM programs in the cities of Pereira and Manizales.

As part of the residency selection process, similarly to that of other medical specialties, applicants take a general medicine written exam and, based on their score, are granted an interview, which may entail an oral examination of medical knowledge, case simulations, and an English language proficiency exam. Most applicants come from major cities including Bogota, Medellin, and Cali. Class size in the different programs ranges from 3 to 7 residents per year. Currently, only one of the programs, University of Antioquia, offers free tuition. The rest charge per semester the equivalent of approximately US$ 6,000. The curricula of the different programs come mostly from the American EM training programs and ABEM model, though with significant variability among programs. EM resident training in bedside point-of-care ultrasound is lacking, but there is significant interest from trainees and the programs. All programs have a research requirement but there is a limited EM faculty development and mentorship available to the residents. Given that emergency medicine as a specialty has only been existence for a relatively short period of time, there has been low resident enrollment and some attrition, with the downstream effect of most residency programs suffering from a limited number of emergency medicine-trained academic faculty. Albeit the opening and expansion of residency programs across the country, emergency medicine remains a new specialty in Colombia, with a small critical mass of emergency physicians practicing clinical medicine in the community and teaching in the residency programs.

The current qualification to become an EM specialist is completion and graduation from an approved EM residency program. Most EM specialists currently practice in major academic teaching hospitals affiliated with an EM residency program. In a few major teaching hospitals without EM residency programs, EM specialists provide care to high acuity patients and supervise the care provided by the general practitioners to lower acuity patients. In general, the EM specialists have a broad scope of clinical practice with focus on the acutely ill medical and surgical patients who arrive to the resuscitation areas in the emergency department. There are several EM specialists in leadership positions who are currently working as chairs, chiefs, medical directors, assistant directors, and program directors in several emergency departments.

Despite these advancements in training and leadership, there was a major setback to the national efforts to get hospitals to staff the departments with EM specialists. Part of the 1414 law stated that EM trained physicians should be the first choice of physician to staff the emergency departments in hospitals providing higher level of care. However, this portion of the law was removed in May of 2013, seriously threatening the demand, income, and recognition of the specialty. Similarly, several institutions have granted the title of EM specialist to physicians who did not complete an emergency medicine residency. The significant shortages and now inequality in qualifications of EM specialists across the country may significantly impact the quality of care delivered to patients and potentially create a national healthcare and public health dilemma.

### Emergency medicine society

The Asociacion Colombiana de Especialistas de Emergencias (ACEM) stands currently as the recognized EM organization in the country. It has achieved full International Federation of Emergency Medicine (IFEM) membership. In 2010, ACEM organized and sponsored its first international EM congress in Bogota marking an academic milestone for the development of academic EM in Colombia. In 2012, the congress expanded its international speaker attendance and audience reach. Despite ACEM’s accomplishments, there are ongoing challenges with membership recruitment and retention. Currently, a core leadership group is continuing to maintain the fledgling organization and attempting to reinvigorate its membership.

## Discussion

The critical challenges for EM are a lack of academic development and recognition of the specialty and a need for a critical mass with unity among the EM specialists. First, the small core of academic EM faculty are working a full clinical load and living cities where the cost of living necessitates a focus on clinical salary. Many of them also do not have the adequate training and preparation for rigorous research and work in departments with limited funding and resources. These faculty members are in a dire need for protected time to be able to teach the clinical practice of emergency medicine, as well as, learn the tools and dedicate their time to publishing scholarly work. Secondly, the lack of a critical mass of EM specialists stems from the low number of students entering the residency training programs and the attrition of potential faculty to lucrative jobs in the ICU. Since it is still a new specialty, most medical students are accustomed to emergency departments being staffed by mostly general practitioners. Only with time and exposure to EM faculty will the students and general practitioners working in emergency departments begin to see the benefit of residency training and acquisition of advanced skills. Lastly, complicating the current situation of a small number of EM specialists in the country are the separation of smaller groups by regions, mainly Bogota and Medellin, and the lack of a national unity among both the EM specialists and residents within ACEM and its leadership structure. A unified specialty will be necessary to be able to address the major issues facing the specialty.

Recognizing that EM is a nascent specialty and its maturation and dissemination across the country will likely take decades, as it did in the US, current EM physicians face the challenge of providing the highest quality and safest emergency care today to the people of Colombia in the current circumstances. There are several key stakeholders or lever points that need to be addressed in order for a successful campaign to change the culture about emergency medicine. At the medical school level, medical students need to have a basic understanding and capability to care for and stabilize an acutely emergent patient that they will encounter during their rural year. In fact, most of these students when they graduate will become general practitioners who will care for the majority of emergency patients in the short term. Every medical school should address this in its curriculum, along with medical simulation training for students. As medical students begin to explore potential careers, there should be an aim to have an EM interest group in every medical school with an EM faculty sponsor. At the residency level, there needs to be a substantial growth and support of EM residencies based in each major city and affiliated with the major teaching hospitals to ensure an adequate supply of emergency physicians to meet the current demand. Residency leadership at each institution should continue its efforts of producing highly competent emergency physicians who can work clinically in any environment, teach students and residents of all specialties, and perform clinical research. Currently, these programs have robust educational curricula coupled with assessment programs that monitor resident progress and ensure adequate promotion. For the last 10 years, these residency programs have set the standard that newer programs will follow. There should be dedicated and concerted effort by major teaching hospitals and affiliated medical schools in each major city to open enough residency programs in order to produce a critical mass of practicing physicians and faculty members within the next 10 to 20 years. As in all specialties of medicine, a strong faculty development program will need to be in place for the growth and advancement of the teaching faculty, as they learn how to teach and advance the science and clinical practice of emergency medicine. The organization of emergency medicine is an important point with respect to the country-specific certification standards to which the specialist will hold each other accountable to and ultimately grant board certification to each specialist. Equally important is the decision to allow or not any other nonresidency trained physicians into the specialty through a ‘grandfathering’ clause similar to what was allowed in the US. This controversial issue gets to the crux of the dilemma faced by many countries alike, one of granting certification to physicians who have neither undergone nor paid for 3 to 4 years of rigorous specialized training. The importance of the emergency department and the specialists with regard to public health, surveillance, and disaster response and preparedness cannot be overstated. The emergency department is the door to the public, and any infectious outbreak will be signaled by a sharp increase in volume in that medical condition. Along the same lines, the emergency physicians are the ideal providers to lead a disaster response, as evident in the teams deployed to Haiti and around the world, given their multidisciplinary skill set and triage capabilities and thus have much to contribute in a country so susceptible to natural disasters like Colombia. Lastly, the public should be educated about the importance of the emergency medicine specialty and its positive effects for the healthcare delivery in Colombia, through as many public media outlets as possible. This will ultimately lead to the public’s demand for emergency physicians and high level of care in the emergency department. The challenges remain daunting for the specialty, but a bright and hopeful future is in the hands of a passionate and dedicated group of specialists in Colombia, driven by the mission to provide the best care that Colombians deserve.

## Conclusion

Colombia is uniquely positioned as an upper-middle-income country with a population of 45 million people and one of the best national healthcare and medical education systems in South America. Despite its challenges between urban and rural settings, the delivery of emergency care is evolving as the specialty of emergency medicine is maturing. Supported by some of the strongest academic institutions of higher education in the major cities of Colombia, residency programs are producing first-class academic emergency medicine specialists. While critical challenges for EM in Colombia include a lack of academic development to ensure recognition of the specialty and a need for a critical mass with unity among the EM specialists, EM is a nascent specialty that will likely take over 30 years for its maturation and dissemination across the country in order to provide the highest quality and safest emergency care today to the people of Colombia.
